# Clinical evaluation of novel blood collection and storage bags containing alternative plasticizers to DEHP: Recommendations from the BEST collaborative

**DOI:** 10.1111/trf.18470

**Published:** 2025-10-29

**Authors:** Bethany Brown, Thomas R. L. Klei, Tamir Kanias, Dirk de Korte, Jose A. Cancelas, Rebecca Cardigan, Biomedical Excellence for Safer Transfusion (BEST) Collaborative

**Affiliations:** ^1^ American Red Cross, Biomedical Services, Medical and Scientific Office Washington DC USA; ^2^ Department of Product and Process Development Sanquin Blood Supply Amsterdam The Netherlands; ^3^ Vitalant Research Institute Denver Colorado USA; ^4^ Hoxworth Blood Center Cincinnati Ohio USA; ^5^ Dana‐Farber Cancer Institute, Harvard Medical School Boston Massachusetts USA; ^6^ NHS Blood and Transplant Cambridge UK; ^7^ Department of Haematology University of Cambridge Cambridge UK

## 1 INTRODUCTION

Di‐(2‐ethylhexyl) phthalate (DEHP) has been used for nearly 70 years to produce flexible blood bags for whole blood (WB) and red blood cell (RBC) storage. The European Union (EU) Commission and European Chemicals Agency have been moving toward a ban of DEHP plasticizer, due to concerns regarding its potential toxicity including the incineration of waste plastic in the environment.^1^ The Registration, Evaluation, Authorisation, and Restriction of Chemicals (REACH) regulations now stipulate that from July 1, 2030, there will be no exemption for the manufacture in the EU and United Kingdom of any medical devices containing DEHP, including blood bags. Some manufacturers of blood bags may make this transition earlier.^2^, ^3^, ^4^ In parallel, the Medical Device Regulations (MDR) are replacing the Medical Device Directives (MDD) in the EU.

Because of the possible impact on the blood supply, cross‐organization coordination is crucial.^5^ There are numerous key stakeholders in the effort to eliminate DEHP from blood bags: blood bag manufacturers, blood establishments, regulators, hospitals, and patients. Blood bag manufacturers are responsible for designing new DEHP‐free blood bag kits and generating evidence on the new bag systems for CE mark and other regulatory approvals. A Blood Transfusion Alliance has been formed in response to the requirement to eliminate DEHP in blood bags and it includes many blood bag manufacturers working together to comply with these new requirements.^3^

The European Blood Alliance (EBA) released guidance in February 2022 on recommendations for in vitro evaluation of blood components collected, prepared, and stored in DEHP‐free medical devices.^6^, ^7^ Complementing this, the European Good Practice Guideline to Authorization on Preparation Processes in Blood, Tissues, and Cell Establishments (GAPP) proposes risk‐based evaluation criteria for blood components (Part 2) and leverages the EuroGTP II risk assessment tool to facilitate the authorization of substances of human origin (SoHO).^8^ While the in vitro evaluation criteria are well defined in this document (Section 3.2), manufacturers need to determine how to cross‐leverage data for multiple bag types. Section 4 of the GAPP discusses assessment of clinical data which may come from clinical investigations, literature, or real‐world data/clinical experience and it is the responsibility of the bag manufacturer to generate. There is a lack of guidance on the type and extent of pre‐market *clinical* data required to ensure safety and efficacy according to EU Guidelines, and currently no food & drug administration (FDA) published acceptance criteria for the assessment of alternatives to DEHP. There is an urgent need to bring together medical, scientific, and operational experts in blood component production and storage to consider this.

The BEST Collaborative has therefore developed recommendations related to the in vivo data required in relation to the removal of DEHP plasticizer from blood bags to support alignment of blood bag manufacturers, regulators, and blood centers. This alignment will facilitate cross‐leveraging data to support new DEHP‐free blood bag systems and decrease risks to the blood supply associated with the burden of evaluating the DEHP‐free systems. Here we make science‐based recommendations on the level of clinical data required prior to the implementation of alternatives to DEHP, and the division of responsibilities for providing these data between the key stakeholders; we are not considering changes from the MDD to MDR or up classification of devices. Since there is currently a hard deadline on the removal of DEHP from blood bag manufacturing in the EU, we need to balance ensuring that alternatives to DEHP are appropriately validated prior to use while ensuring that this burden is manageable for all stakeholders in order to mitigate the risk of failure to supply blood bags and blood for transfusion.

## 2 MEDICAL DEVICE/BIOLOGIC MANUFACTURERS AND PRODUCT MANUFACTURERS RESPONSIBILITY

Elimination of DEHP is one of three upcoming regulatory requirements in the EU impacting blood bags/products; these topics must not be confused, and blood bag manufacturers must consider all three changes in the CE mark updates including: (1) updated EU MDR from MDD (EU 2017/745), (2) elimination of DEHP (EU 2023/2482), and (3) up‐classification of bag systems containing anticoagulants/additive solutions (EU 2017/745).

Our aim is to provide recommendations for in vivo testing specifically for DEHP‐free blood bag systems. However, it is ultimately up to blood bag manufacturers to demonstrate conformity with the relevant general safety and performance requirements (GSPRs) in the field of SoHO for their blood bags in consultation with relevant regulators. The evidence compiled on blood bags will be leveraged in the technical file/design dossier to allow distribution of new bags to blood establishments for blood product manufacturing. There are three main data considerations for evidence generation to support the CE mark of new non‐DEHP blood bags for all blood product types: pre‐market *laboratory studies*, pre‐market *clinical* data, and post‐market surveillance (PMS) including post‐market clinical follow‐up (PMCF).^6^, ^9^ Additionally, bag manufacturers will follow standard requirements for testing biocompatibility (e.g., hemocompatibility, cytotoxicity, sterility, and irritation), physical and chemical testing (e.g., leachable, bag integrity), and performance and stability testing; these categories of bag testing are out of the scope of this discussion. Blood bag manufacturers must work with regulators to define the studies needed to demonstrate safety and efficacy based on the risk–benefit assessment of each blood bag/product. EuroGTP II is an interactive assessment tool that can be used to identify, quantify and assess the level of risk associated with novel SoHO preparations.^10^ Blood bag manufacturers must also define PMS and PMCF plans based on existing evidence and benefit–risk assessment as well as assessments of the acceptability of the benefit–risk ratio throughout the lifecycle of the marketed product.

Blood operators, on the other hand, must validate new bags for blood center licensing and preparation authorization and can support evidence generation through user experience, enhanced hemovigilance, or additional product quality studies. In addition to conducting studies to support bag licensing of the primary storage bags, blood centers and regulators will require assurance that downstream secondary steps in blood processing such as washing, irradiation, pathogen reduction, or other downstream steps in blood processing (e.g., freezing and deglycerolizing, cryoprecipitate production) on the investigational storage bags do not have unexpected effects on blood quality.

## 3 EFFECT OF DEHP ON BLOOD COMPONENTS AND RECOMMENDATIONS

During their storage, cellular blood components such as RBCs and platelet concentrates (PCs) steadily undergo changes that impact their quality. Metabolic decline, activation, expression of apoptotic and inflammatory markers, morphological changes, cellular disintegration, and adhesion molecule activation are collectively known as the storage lesion. The extent of the lesion is dependent on factors including storage medium, storage duration, temperature, processing steps, and donor‐specific factors. Moreover, the exposure of the overall population, including blood donors, to environmental stimuli such as pollutants and drugs, the choice of certain behaviors including food preferences, smoking and alcohol consumption, and the presence of a specific microbiome might impact WB, RBC, platelet, and plasma quality.^11^

It has also long been known that the plasticizer used in blood collection devices can profoundly impact blood product quality. It is well established that DEHP, a plasticizer that ensures flexibility and integrity of blood bag sets, is non‐covalently bound to the polyvinyl chloride (PVC) and as such can leach into blood products. Due to its lipophilic nature, it is also incorporated into cellular membranes. Although PCs are unaffected by this incorporation, RBC membranes are stabilized by this plasticizer.^12^, ^13^ The main effects on RBC storage in the absence of DEHP are increased hemolysis, microparticle generation and osmotic fragility, reduced deformability, but have limited impact on metabolic parameters such as adenosine triphosphate (ATP) and 2,3‐diphosphoglycerate (2,3‐DPG).^14^, ^15^, ^16^ To our knowledge there is no impact of this change on RBC antigenicity.

Now that DEHP is being banned from use in medical devices, alternative plasticizers are being explored. Initial results showed a detrimental effect in RBCs stored in bags made with diisononyl cyclohexane‐1,2‐dicarboxylate (DINCH; in SAGM additive solution) or with di(2‐ethylhexyl) terephthalate (DEHT) plasticizer (stored in Additive Solution‐1 [AS‐1]), reported increased hemolysis.^9^, ^17^ However, additional promising results have been obtained in DINCH, DEHT, and butyryl‐tri‐hexyl‐citrate (BTHC)‐containing storage bags in combination with the second‐generation storage solution PAGGSM, AS‐3, and third‐generation storage solution AS‐7, yielding hemolysis levels comparable to the current, DEHP‐containing, RBC products.^9^, ^17^, ^18^, ^19^, ^20^, ^21^ These advancements render a non‐DEHP future possible, in which product quality remains equivalent to currently approved RBC products.

There is no similar evidence that PCs and plasma are affected by DEHP or its absence.^20^, ^21^ For many years, PCs have been stored in bags that do not contain DEHP, using alternative plasticizers such as BTHC and trioctyl trimellitate (tris(2‐ethylhexyl) trimellitate) (TOTM), although some parts like tubing and ports often contain DEHP. Furthermore, the European Pharmacopeia already allows plasticizers such as DINCH, BTHC, DEHT, and TOTM to be used to manufacture plasma and platelet storage bags.^22^ The question remains as to which components are to be evaluated in vivo and to what extent. No clear direction is provided by currently available European legislation or the FDA. Unexpected adverse events are not anticipated, due to the extensive laboratory biocompatibility studies required to be undertaken as part of regulatory approval. This paper makes recommendations on the in vivo evaluations needed to assure quality of the blood collected and stored in non‐DEHP systems, based on the best available scientific evidence.

### 3.1 Recommendation and rationale for in vivo evaluation of platelets/granulocytes

Platelet interaction with DEHP is relatively low when compared to RBCs, resulting in low concentrations of DEHP in PCs.^23^ Several studies have found no meaningful impact on platelet quality parameters, recovery, survival, or hemostatic effectiveness.^20^, ^21^, ^24^ PCs are already stored in PVC‐TOTM and PVC‐BTHC storage bags, which provide superior oxygen permeability and help preserve platelet quality during storage. Because of this extensive clinical experience, pre‐clinical in vivo evaluation of non‐DEHP PC is not warranted. Unlike RBCs, DEHP is not protective for leucocyte function. On the contrary, DEHP has been associated with a reduction in markers of neutrophil function such as chemotaxis and bacterial killing,^25^, ^26^, ^27^ Therefore the removal of DEHP is likely to have beneficial rather than detrimental effects and it is proposed that laboratory testing be limited to an operational validation to assess the impact of the change of plasticizer as recommended by the EBA.^6^

### 3.2 Recommendation and rationale for in vivo evaluation of plasma

Plasma for transfusion is usually stored frozen, limiting the opportunity for DEHP leaching except during the pre‐freeze and post‐thaw phases. Available data demonstrate no significant effects of DEHP on plasma quality parameters, including immunoglobulins and clotting factors.^6^, ^15^, ^20^, ^21^ Although different plasticizers may interfere with some plasma protein analyses, this does not suggest a biological impact.^9^, ^24^, ^28^ Based on accumulated in vitro evidence, pre‐clinical in vivo evaluation of plasma is unnecessary. Risk classification using the EuroGPTII risk assessment tool would likely rate these products as negligible to low risk. Instead, hemovigilance programs should be used to monitor for any increase in the frequency or severity of rare events such as allergic reactions which are not detectable in small‐scale studies.

### 3.3 Recommendation and rationale for in vivo evaluation of RBCs

Among all blood components, RBCs remain the primary concern during the transition away from DEHP due to the stabilizing effect of the plasticizer on RBC membranes.

**FIGURE 1:**
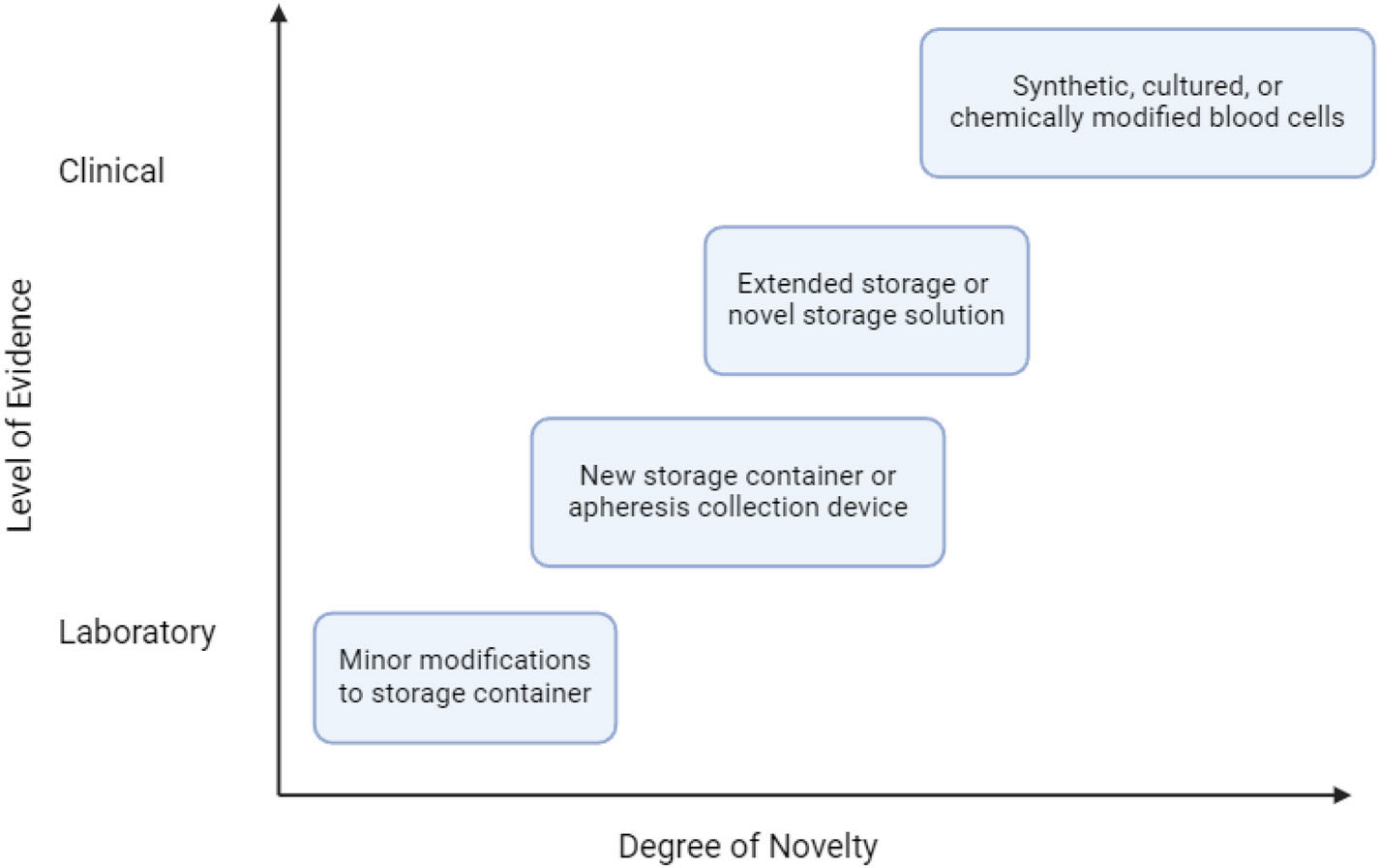
Hierarchy of studies to evaluate changes to red blood cells (modified as previously described for platelets from Vostal, 2006).

For changes to RBC storage, there is a hierarchy of studies that are normally performed to ensure RBCs remain viable, the magnitude of which depends on the nature of the change and applicable regulatory framework (Figure 1).^29^ This ranges from solely laboratory studies for low‐risk changes, to studies in an appropriate patient population for major changes. While this has historically varied across jurisdictions, the upcoming implementation of the EU implementation of the SoHO regulations will harmonize regulatory oversight across EU Member States and adopt a risk‐based framework, potentially standardizing requirements for such studies to support the CE mark. However, global requirements may continue to vary (e.g., US FDA). For decades, 24‐h recovery after transfusion of a small dose of radiolabeled autologous RBCs into healthy volunteers has been used as a surrogate marker for RBC efficacy in patients, with the assumption that if RBCs can circulate, they will also function. There is traditionally no requirement to measure longer‐term survival, since most effects are seen within 24 h of transfusion, and survival curves are parallel thereafter. 24‐h recovery studies are predominately performed in the United States, because the US FDA requires these studies. Despite the fact that several sites in the EU can perform such studies, they have not been required by EU regulators to grant CE mark for recent changes to RBC storage such as new RBC storage solutions (e.g., PAGGSM), new blood bags, and automated/semi‐automated blood processing devices (e.g., Reveos) now in routine use. Although the FDA requires in vivo studies for significant changes to RBC storage conditions (e.g., the additive solution), they have not yet defined which in vivo studies would be required to license blood bags with alternative plasticizers to DEHP and neither have EU regulators, although regulatory requirements may not be the same globally.

Studies comparing DEHP‐PVC with TOTM‐PVC have shown higher in vitro hemolysis and reduced in vivo RBC recovery (<75%) in the TOTM group.^14^, ^30^, ^31^ The authors suggest that this effect may be due to the changes in RBC membrane.^32^, ^33^ AuBuchon et al. evaluated the effect of DEHP and RBC recovery and survival and found that while in vitro analysis differences were modest, including increased hemolysis, in vivo recovery and survival was higher in the DEHP‐stored RBCs compared to non‐DEHP storage containers.^14^ Two other studies evaluating BTHC‐plasticized PVC found in vivo recoveries >80%, although hemolysis was somewhat higher than in DEHP‐PVC.^34^, ^35^ We are not aware of any studies where new plastics have resulted in comparable hemolysis levels, yet poor in vivo recovery. There does appear to be a relationship between RBC ATP and RBC recovery in vivo, and this has been used as an argument that provided ATP levels are >50% of fresh values, RBC recovery may be adequate^33^, ^36^, ^37^, ^38^, ^39^; however, aggregate data suggest that in vitro data cannot be used to predict in vivo recovery data. Further, in the case of alternative plasticizers, this is unlikely to be helpful, since it is well known that these impact RBC morphology, deformability, osmotic fragility, microparticle release and RBC survival without affecting key RBC metabolic parameters such as ATP and 2,3‐DPG.^14^

The goal of blood operators and blood bag manufacturers is to make the transition to non‐DEHP as invisible as possible to hospitals and patients by combining new plasticizers with well‐known storage solutions already in use, in order to maintain RBC quality to as close to current as possible. In these circumstances, where the laboratory data on new plasticizers appear similar to that already in use, there are three key concerns that should be mitigated. First, there could be an unexpected reduction in RBC recovery and survival or function although the laboratory data are acceptable. Based on the data to date this appears very unlikely. Second, there may be unexpected adverse events such as allergic reactions to RBC/platelets/plasma components—these are unlikely to be seen in small‐scale autologous studies of a mini‐dose of RBC/platelets in healthy volunteers. Third, it is unknown if this change will impact the rate of bag failures, leaks, and other routinely used metrics.

Although in vitro and in vivo RBC studies are independent evaluations of each other and one evaluation may not rule out the need for the other,^14^ a change in plasticizer alone does not warrant pre‐clinical in vivo evaluations. Additionally, key parameters of interest on the effect of alternative plasticizers to DEHP, such as impact on transfusion efficacy or safety, are unlikely to be observed in small‐scale studies in healthy volunteers. Thus, we recommend using the published EBA recommendations to evaluate comprehensive laboratory data that characterizes different facets of RBC structure and function.^6^ Provided a robust in vitro dataset demonstrates comparable in vitro product quality compared with the current standard, 24‐h recovery studies are unlikely to be informative and should not be an absolute requirement to proceed to a controlled implementation following validation for change in a blood bag plasticizer alone. Importantly, it will be critical to combine extensive laboratory validation with PMS at scale to assess any unexpected or infrequently observed events following implementation.

In line with the paper published by EBA, not only is it important to gather ample in vitro data for primary storage of RBCs, but also for components that have undergone secondary processing steps.^6^, ^9^ The additional in vivo evidence required will depend on the extent of the change to RBCs observed during primary processing, in addition to the well‐known effects of processing steps such as irradiation and washing which can have a detrimental effect of RBCs. Currently, the effect of washing with non‐DEHP bags has not been studied and one study demonstrated that irradiation of RBCs DEHT/PAGGM was comparable to DEHP/SAGM and remained within acceptable quality limits.^40^ Ultimately, it is important to consider whether the combination of changes is more than additive. For example, if non‐DEHP bags are shown to be non‐inferior to the current standard of care, there is no rationale for additional clinical data on such cells that subsequently are subjected to current standard and well‐characterized secondary processing steps. Additionally, it would be virtually impossible to assess all the different possible combinations of primary and secondary processing in clinical studies. As for standard RBCs, post‐marketing hemovigilance/PMS programs should be structured to capture secondary processing data.

Although outside the immediate scope of plasticizer replacement, additive solution composition significantly impacts RBC preservation and shelf‐life. The up‐classification of medical devices under MDR primarily concerns anticoagulants and additive solutions, not plasticizers. Therefore, RBCs stored in non‐DEHP bags with next‐generation additive solutions should undergo laboratory validation and PMS to ensure sustained product potency and safety.

### 3.4 Recommendation and rationale for in vivo evaluation of WB

Transfusion of WB is resurging with retrospective studies and meta‐analyses suggesting that the administration of WB in trauma and major hemorrhage is non‐inferior, and in some cases may even be more beneficial, to component therapy.^41^, ^42^, ^43^, ^44^, ^45^, ^46^ Although much is already known about the impact of non‐DEHP on the individual components, this is not the case for WB, especially when it concerns the interaction between the components and non‐DEHP plasticizers. However, differences in RBC recovery when stored in PVC‐TOTM compared with DEHP were not lower when stored as WB than when stored as RBCs (hematocrit 75%) in CPDA‐1.

One study comparing PVC‐TOTM with DEHP‐PVC in CPDA‐1 showed acceptable RBC recovery up to 21 days, consistent with the protective effect of DEHP being most evident during prolonged storage.^31^

Although WB is typically stored for shorter durations and with different anticoagulants, data from RBCs can reasonably be extrapolated to WB. Therefore, the evaluation of non‐DEHP WB should include robust in vitro assessments of RBC, plasma, and platelet parameters, supplemented by PMS to identify any rare or unexpected issues.

## 4 ASSESSMENT OF IN VIVO RECOVERY IF NON‐DEHP IS COMBINED WITH OTHER SIGNIFICANT CHANGES

The regulatory requirement for these types of studies to validate novel RBCs varies by jurisdiction, although EU Regulation on SoHO will harmonize regulatory oversight across EU Member States. In our view, with respect to the move away from DEHP, in vivo studies would only be warranted if there is also a very novel type of plastic, unlicensed additive solution, or in combination with an extension in shelf‐life (Figure 1). If these studies are performed, radiolabeling of blood cells remains the gold standard method to evaluate 24‐h post‐transfusion RBC recovery in an autologous healthy donor clinical study. There is a well‐defined clinical study structure and endpoint for radiolabeling studies which includes at least 20–24 normal healthy research participants performed at two sites (minimum) to show a mean value for the in vivo RBC recovery at 24 h of ≥75%, with a standard deviation of ≤9% and a 95% one‐sided lower confidence limit for the population proportion of successes ≥70%.^47^, ^48^ This statistical criterion is based on historical evaluation of many radiolabeling studies. Such studies are permitted in only a few jurisdictions (such as the United States and United Kingdom); however, feasibility studies have shown success with using biotinylated RBCs as an alternative to radiolabeling.^49^, ^50^, ^51^, ^52^ Given the regulatory hurdles for radiolabeling studies, a biotinylated RBC method has been investigated for in vivo efficacy evaluations^53^; however, any new method for RBC recovery and survival studies should have a similar standard deviation, or the sample size may need to be recalculated and discussed in public and scientific forums and with regulators, along with acceptance criteria and data on antibody formation.

## 5 HEMOVIGILANCE AND POST‐MARKET SURVEILLANCE

MDR require that manufacturers establish PMS and PMCF plans (Annex III of MDR).^54^ When the CE mark is based on equivalence, PMCF studies may be necessary and may leverage hemovigilance systems. Furthermore, if a legacy device does not have sufficient post‐market data, new data may be needed. PMS should continually re‐verify and re‐validate the results of the development phase with real‐world data, to demonstrate conformity to the MDR GSPR for the entire life cycle of the product. Benefit–risk determinations should periodically be updated to improve the product's risk. Many existing hemovigilance systems for blood are focused on adverse events related to transfusion including categories such as allergic and febrile reactions, hemolytic reactions, and so forth. Existing systems are not designed to capture data on the efficacy of the component. Following the transition to non‐DEHP blood bag systems, we recommend enhancing hemovigilance efforts by collecting data on relevant parameters (Table 1), where appropriate. It is also recommended to collaborate across the blood industry to create hemovigilance databases to capture data by product (including anticoagulant type, bag name, additive solution, etc.) to help cross‐leverage data for multiple users, if it is possible to share de‐identified data across organizations. Additionally, if enhanced hemovigilance data are planned to be collected, *it is recommended that this begins prior to* the *introduction of non‐DEHP kits to provide data on historic controls*. Importantly, in addition to the MDR which stipulate the need to conduct PMS, there is scientific and clinical justification to generate robust hemovigilance data preferably with sufficient power to be able to claim non‐inferiority for relevant endpoints and provide reassurance at scale that this transition has not resulted in any unexpected effects.

**TABLE 1:** Parameters to consider for blood bag hemovigilance.

Parameter	Product type
Bag/donor‐level data	
Bag integrity (e.g., leaks)	RBCs, platelets, plasma, WB
User issues	RBCs, platelets, plasma, WB
Blood donor adverse events	RBCs, platelets, plasma, WB
Hemolysis	RBCs, WB
Patient‐level data	
Blood recipient transfusion reactions	RBCs, platelets, plasma, WB
Hemoglobin increment	RBCs, WB
Corrected count increment	Platelets
Transfusion intervals	RBCs, platelets
Product utilization	RBCs, platelets, plasma, WB

Abbreviations: RBCs, red blood cells; WB, whole blood.

Of note, DEHP exposure has also been observed among voluntary plasma and platelet donors due to prolonged periods of contact with disposable plastics for collection.^55^ The latter authors observed elevated DEHP levels in blood or urine samples of patients undergoing plasmapheresis, plateletpheresis, and extracorporeal membrane oxygenation treatment. Recently, von Ostau et al. demonstrated that plateletpheresis leads to significant phthalate exposure, which correlates with a temporary and reversible decline in sperm motility.^56^ It is therefore important that as blood operators transition to non‐DEHP, consideration is given to blood donors as well as patients. Manufacturers of apheresis devices are responsible for ensuring aspects related to toxicity are covered during regulatory approval.

## 6 CONCLUSION

In summary, non‐DEHP containing blood bags for the collection and storage of WB, RBCs, platelets, plasma and granulocytes can be adequately evaluated for safety and efficacy with a panel of appropriate pre‐clinical laboratory tests. In vitro evaluation should follow GAPP (Facilitating the Authorization of Preparation Process for Blood, Tissues, and Cells) recommendations released in 2022 and accepted by members of the EBA.^6^, ^8^

Pre‐market clinical studies are not warranted, as this expert group considers the risk to be low and these will not add critical data to the decision‐making process. This applies to a change in plasticizer alone with established plastics, or in combination with anticoagulants in current use for blood collection and/or storage or licensed newer generation additive solutions. Anticoagulants, very novel additive solutions, and plastic storage bags that are not in use or licensed will likely require additional pre‐clinical evaluation. Well‐thought‐through hemovigilance/PMS data collection that can differentiate different types of products will be essential to detect any unexpected low‐frequency adverse reactions as a result of this change once implemented. Manufacturers will need to work with regulators, whose requirements may vary globally, to confirm that the totality of changes to their products is supported by the existing totality of data and proposed additional data generation plan. These are the next steps necessary as we work toward the implementation of DEHP‐free blood production.

## CONFLICT OF INTEREST STATEMENT

BLB, TRLK, TK, and RC have no relevant conflicts of interest to declare. JAC is a consultant for Cellphire Inc., Velico, Hemanext Inc., Terumo BCT, Fresenius‐Kabi Inc., Teleflex Inc., Preservation Bio and Rion. He has received grant support from Velico, Terumo BCT, Cerus Co, and Cellphire Inc. He also owns equity in Preservation Bio and intellectual property in Platefuse Inc.

## Data Availability

Data sharing not applicable to this article as no datasets were generated or analysed during the current study.
